# Implicit and explicit systems differently predict possible dangers

**DOI:** 10.1038/s41598-019-49751-4

**Published:** 2019-09-16

**Authors:** Eugenio Manassero, Ludovica Mana, Giulia Concina, Annamaria Renna, Benedetto Sacchetti

**Affiliations:** 1 Rita Levi-Montalcini Department of Neuroscience, University of Turin, Corso Raffaello 30, I-10125 Turin, Italy; 2 National Institute of Neuroscience, Turin, Italy

**Keywords:** Human behaviour, Fear conditioning

## Abstract

One strategy to address new potential dangers is to generate defensive responses to stimuli that remind learned threats, a phenomenon called fear generalization. During a threatening experience, the brain encodes implicit and explicit memory traces. Nevertheless, there is a lack of studies comparing implicit and explicit response patterns to novel stimuli. Here, by adopting a discriminative threat conditioning paradigm and a two-alternative forced-choice recognition task, we found that the implicit reactions were selectively elicited by the learned threat and not by a novel similar but perceptually discriminable stimulus. Conversely, subjects explicitly misidentified the same novel stimulus as the learned threat. This generalization response was not due to stress-related interference with learning, but related to the embedded threatening value. Therefore, we suggest a dissociation between implicit and explicit threat recognition profiles and propose that the generalization of explicit responses stems from a flexible cognitive mechanism dedicated to the prediction of danger.

## Introduction

Encountering a novel stimulus demands an organism to predict its emotional implications to properly react. A possible strategy is to generate defensive responses to stimuli that remind an individual of threatening events, a phenomenon called fear generalization^1–4^. Stimuli perceived as dissimilar from a threat can be detected as neutral or not dangerous, thus eliciting a discriminative response. Survival requires an adaptive balance between generalization and discrimination^1,5^. When this delicate mechanism undergoes dysregulation, the resulting behavior can be maladaptive, and overgeneralization has been proposed as a pathogenetic marker of the anxiety disorders spectrum^6–10^. Hence, understanding which mechanisms underlie threat identification would represent a fundamental advance to approach pathologies such as posttraumatic stress disorder, panic disorder and generalized anxiety disorder.

According to a recent conception^5^, fear generalization is an active process that arises from the integration of signals deriving from two sources of information, a threat-identification mechanism and an ambiguity-based uncertainty-evaluation mechanism. The tuning of behavioral and autonomic responses similarly adjusts to a perceptual gradient, and these responses could be predicted by combining these representations rather than being passively shaped by physical resemblances^5^.

During a threatening experience, the human brain encodes implicit and explicit memory traces that are mediated by different neural circuits^11,12^. In this framework, fear generalization dynamics have been characterized by recording implicit autonomic indicators (for example, skin conductance responses, SCRs). Some studies have employed US-expectancy ratings (for example^5,13,14^) or episodic recognition^15^ as explicit parameters of fear expression. However, there are relatively few studies reporting a direct comparison between implicit and explicit gradients in fear generalization (e.g.^5,8,13,16^). Thus, whether explicit generalized behavior is mirrored by a defensive response at the implicit level or whether these two patterns diverge is far from being defined. In other words, whether fear generalization arises from an active process that yields an integrated implicit-explicit outcome, or alternatively, it originates from the activity of multiple systems that trigger dissociable reactions in response to an incoming stimulus is poorly understood. In this study, we intended to address this issue.

## Results

### Different implicit and explicit behavioral responses to novel stimuli

**Figure 1:**
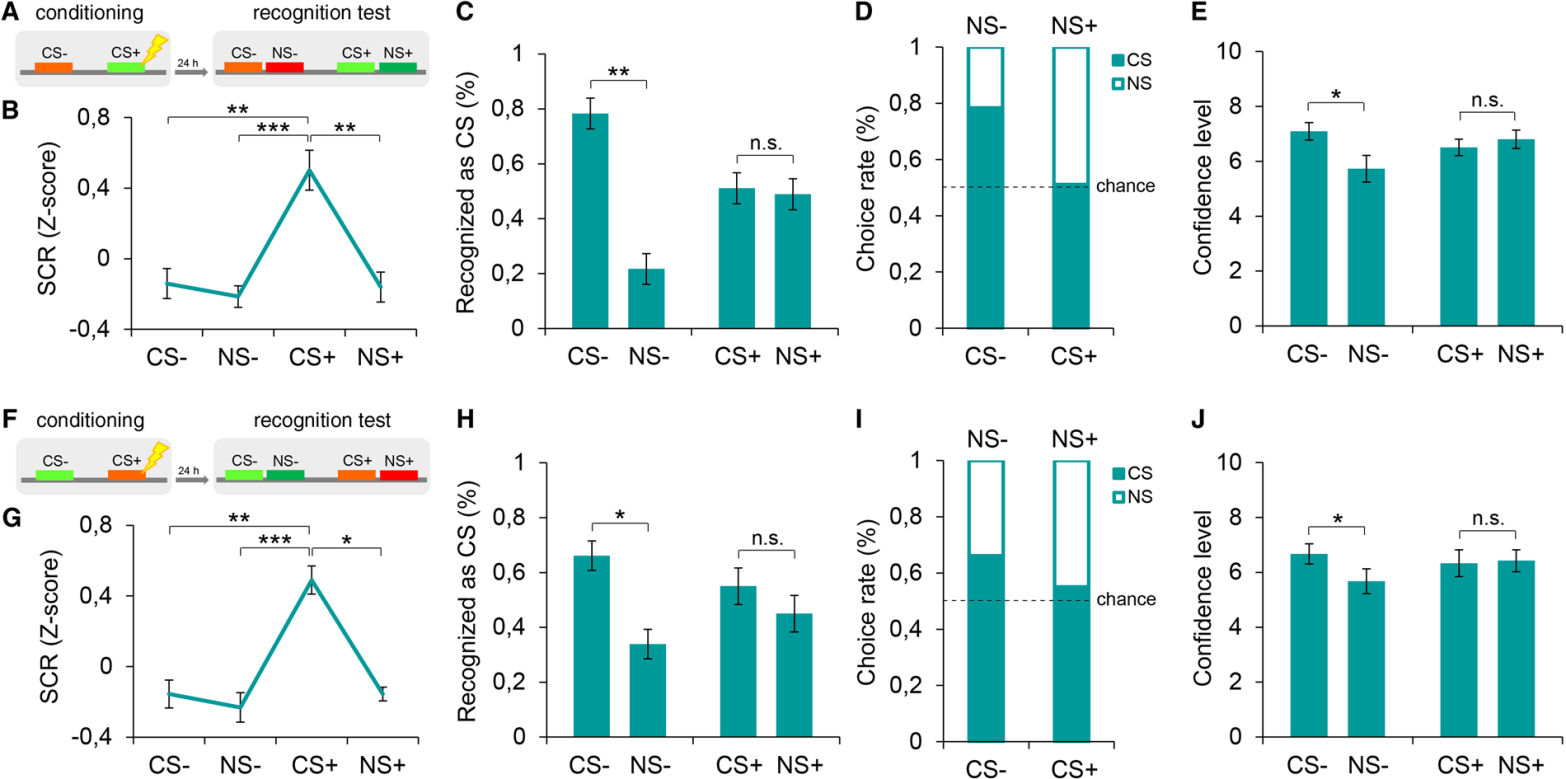
Implicit and explicit threat recognition profiles. (**A**) Schematic diagram depicting the experimental procedures. Participants underwent a discriminative threat conditioning in which a conditioned tone (CS+, 370 Hz) was paired with a mild electrical shock (US) and a non-reinforced tone (CS−, 784 Hz) was never paired with the US. Twenty-four hours later, subjects underwent either an implicit or explicit 2AFC recognition task which consisted of tone-pairs composed by a conditioned stimulus (CS− or CS+) and a novel stimulus similar to the CS− (NS−, 1046 Hz) or to the CS+ (NS+, 466 Hz). **(B)** Implicit threat recognition profiles (*n* = 18) demonstrated a high level of discrimination. **(C,D)** Explicit threat recognition profiles (*n* = 18) showed a threat-selective generalization, as subjects almost equally identified the CS+ and misidentified the NS+ as CS. Vice versa, the CS− was correctly detected over the NS−. **(E)** Confidence levels in the explicit recognition task indicated that subjects were more confident when identifying than when misidentifying the CS− but equally confident in the case of the CS+. **(F)** Discriminative threat conditioning and 2AFC recognition test in which the auditory frequencies of CSs and NSs were inverted (CS−, 370 Hz, NS−, 466 Hz; CS+, 784 Hz; NS+, 1046 Hz). **(G)** High specificity of implicit threat recognition profiles for inverted tone-frequencies (*n* = 18). **(H, I)** In the explicit threat recognition test with inverted tone-frequencies (*n* = 18) subjects tended to misidentify the NS+ as CS while correctly detected the CS− as CS. **(J)** Subjects were more confident for correct than incorrect responses for the CS− but not for the CS+. *P < 0.05, **P < 0.01, ***P < 0.001. All data are mean and SEM. Friedman test followed by Dunn’s *post-hoc* tests [(**B,G**)]; Wilcoxon signed-rank tests [(**C,H**)]; Mann–Whitney U tests [(**E,J**)].

To examine the tuning curves of either implicit or explicit fear responses, we adopted a discriminative auditory fear conditioning paradigm in which participants learned to associate a tone (conditioned stimulus, CS+, 370 Hz) with a mild electrical shock (unconditioned stimulus, US, individually calibrated intensity) and another tone (nonreinforced stimulus, CS−, 784 Hz) with the absence of shock. Twenty-four hours after training, we tested the implicit and explicit ability to recognize the encoded stimuli as well as the reactions to novel cues in separate groups. One group (*n* = 18) underwent an implicit two-alternative forced-choice (2AFC) test, in which subjects were presented with a pseudorandom sequence of tone pairs, each composed of a conditioned stimulus (CS+ or CS−) and a novel stimulus similar to the CS+ (NS+, 466 Hz) or to the CS− (NS−, 1046 Hz). NSs were selected to be harmonically and similarly higher-pitched than the CSs. In line with previous studies^5,13,14^, event-related skin conductance responses (SCRs) were recorded for the assessment of autonomic responsiveness. Another group (*n* = 18) underwent an explicit 2AFC task in which participants heard the same sequence of CS−NS pairs used during the implicit test, and they had to identify which stimulus of each pair was the one previously paired with the US (i.e., the CS+) or the one previously learned as not associated with the US (i.e., the CS−). Subjects were also asked to provide a subjective confidence level for each choice, using an analog scale from 0 (completely unsure) to 10 (completely sure). Compared to a one-stimulus-per-trial new/old judgment task, the 2AFC task improves performance by discouraging response biases such as the familiarity-based decision bias^17^. No US shocks were delivered during the implicit and explicit tests (Fig. 1A).

In the implicit task, participants exhibited a highly precise threat identification pattern (Fig. 1B), as SCR levels showed the highest peak to the CS+ (Friedman test, χ^2^_(3)_ = 20.07, *P* < 0.001) and were weaker to the NS+ (Dunn’s *post hoc* comparison, *P* = 0.001), CS− (*P* = 0.008) and NS− (*P* < 0.001) stimuli, which did not differ from each other (*P* > 0.05 in each comparison). We also found no correlation between the magnitude of the autonomic responses to the CS+ and to the NS+ and trait or state anxiety scores assessed with the State-Trait Anxiety Inventory (STAI-Y) scale (see Table S2).

Unexpectedly, the explicit recognition of threatening and novel stimuli displayed a different tuning profile (Fig. 1C,D). In fact, the participants were more likely to misidentify the NS+ as the learned threatening stimulus, and recognition rates approximated the 50% chance level (51.1% of correct CS+ and 48.9% of incorrect NS+ choices; Wilcoxon signed-rank test, *Z* = −0.15, *P* > 0.05). Importantly, the discrimination of the nonreinforced stimulus (CS−) demonstrated higher levels of precision, as participants were able to correctly identify it in most cases (78.3% of correct CS− and 21.7% of incorrect NS− choices; Wilcoxon signed-rank test, *Z* = −3.28, *P* = 0.001). Subjects were more confident when correctly identified than when misidentified the CS− (Mann–Whitney test, *U* = 60.50, *P* = 0.022) whereas they were equally confident when correctly identifying or misidentifying the CS + (Mann–Whitney test, *U* = 143.00, *P* > 0.05) (Fig. 1E). Additionally, in this case, we found no correlation between the explicit threat generalization (CS+ versus NS+ choice rate) and trait or state anxiety levels (see Table S2).

In two subsets of subjects from the Experiment 1 (standard tone frequencies, *n* = 8) and from the Experiment 2 (inverted tone frequencies, *n* = 9), we also tested the ability to perceptually discriminate as different the sensory stimuli employed as CSs and NSs. These participants underwent a 2AFC discrimination task in which we collected binary ‘same or different’ judgments. The accuracy rates of the sensory comparisons were high and comparable for both tones in the Experiment 1 (CS+ versus NS+: 95% ± 2.67 SEM with a mean confidence level of 9.25 ± 0.41 SEM; CS− versus NS−: 100% ± 0.00 SEM with a mean confidence level of 9.49 ± 0.29 SEM; Wilcoxon signed-rank test, *Z* = −1.63, *P* > 0.05) and in the Experiment 2 (CS+ versus NS+: 100% ± 0.00 SEM with a mean confidence level of 9.57 ± 0.16 SEM; CS− versus NS−: 98.9% ± 1.11 SEM with a mean confidence level of 9.75 ± 0.12 SEM; Wilcoxon signed-rank test, *Z* = −1.00, *P* > 0.05), thus ensuring that these stimuli were clearly discriminable.

To explore the possibility that the explicit generalization we observed to stimuli resembling the CS+ but not the CS− may be related to the auditory frequency of the tones employed as CS+ and CS−, we repeated the experiment by inverting the tone frequencies (i.e., CS−, 370 Hz; NS−, 466 Hz; CS+, 784 Hz; NS+, 1046 Hz) (Fig. 1F). In this condition, in the implicit task (*n* = 18), strong autonomic responses were triggered to the CS+ (χ^2^_(3)_ = 23.25, *P* < 0.001) but not to the NS+ (*P* = 0.040), CS− (*P* = 0.002) and NS− (*P* < 0.001) stimuli (Fig. 1G). In the explicit task (*n* = 18), participants failed to detect the threatening stimulus by almost equally choosing the CS+ (55%) and the NS+ (45%) (*Z* = −0.70, *P* > 0.05). Again, the participants successfully identified the nonreinforced stimulus by mostly choosing the CS− (66.1%) over the NS− (33.9%) (*Z* = −2.46, *P* = 0.014) (Fig. 1H,I). The confidence ratings were higher for correct than incorrect responses for the CS− (*U* = 92.50, *P* = 0.045) but not for the CS+ (*U* = 143.50, *P* > 0.05) (Fig. 1J).

These findings uncovered a dissociation between the implicit and explicit response patterns. Implicit reactions exhibited a sharp discrimination profile resulting in the precise detection of the learned threat. In contrast, the test of explicit recognition showed that subjects generalized the memory representation of the learned threat (CS+) to the novel but similar stimulus (NS+), while they discriminated the nonreinforced stimulus (CS−) from the novel but similar stimulus (NS−), irrespective of the tone frequencies.

**Figure 2:**
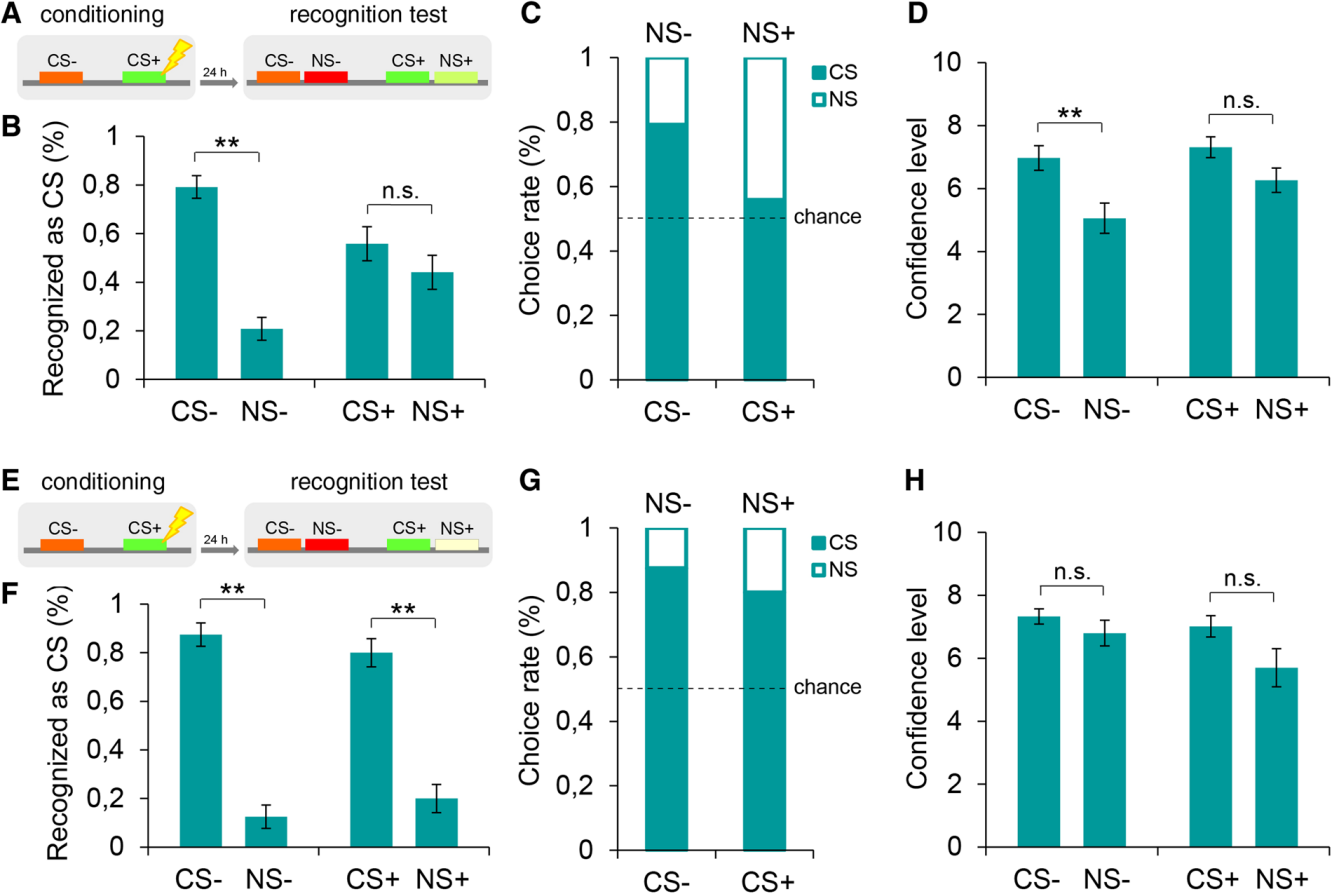
Explicit threat recognition of a symmetric and a distant new tone. (**A**) Discriminative threat conditioning (CS+, 370 Hz; CS−, 784 Hz) was followed, twenty-four hours later, by the explicit 2AFC recognition task with the auditory frequency of the NS+ symmetrically lower-pitched than the CS+ (NS+, 294 Hz; NS−, 1046 Hz). **(B,C)** In the explicit recognition task subjects (*n* = 12) failed to identify the CS+ but correctly detected the CS−. This pattern highlighted a symmetrical two-sided frequency range of the threat-selective generalization of explicit responses. **(D)** Subjects expressed higher confidence levels for correct versus incorrect recognitions of the CS− but not of the CS+. **(E)** Participants underwent a threat conditioning (CS+, 370 Hz; CS−, 784 Hz) and an explicit 2AFC recognition task in which the harmonic distance between the NS+ and the CS+ was doubled, yielding a highly different new tone (NS+, 233 Hz; NS−, 1046 Hz). **(F,G)** Explicit recognition profiles (*n* = 12) indicated that subjects successfully detected both the CS− and the CS+, thus showing a twofold discrimination. **(H)** Subjects were similarly confident when responding to both CSs. *P < 0.05, **P < 0.01, ***P < 0.001. All data are mean and SEM. Wilcoxon signed-rank tests [(**B,F**)]; Mann–Whitney U tests [(**D,H**)].

Next, we investigated the physical boundaries (i.e., tone frequencies) to which the explicit generalization phenomenon could be extended. To this end, we shifted the auditory frequency of the NS+ away from that of the CS+. Because in previous experiments we had adopted a 466-Hz tone as the NS+, to not generate a NS+ that was too similar to the CS− (784 Hz), in one group (*n* = 12), we harmonically reversed the frequency of the NS+ to obtain a tone that was equally distant but symmetrically lower-pitched than the 370-Hz CS+ (i.e., CS−, 784 Hz; NS−, 1046 Hz; CS+, 370 Hz; NS+, 294 Hz) (Fig. 2A). In these conditions, we observed a comparable level of threat-specific generalization behavior in the explicit 2AFC task (Fig. 2B,C). Subjects identified the nonreinforced stimulus (CS−, 79.2% versus NS−, 20.8%, *Z* = −3.08, *P* = 0.002) but failed to recognize the threatening stimulus (CS+, 55.8% versus NS+, 44.2%, *Z* = −0.63, *P* > 0.05). Confidence judgments were higher for correct than incorrect responses for the CS− (*U* = 18.50, *P* = 0.009), whereas no differences were found between the CS+ and the NS+ (*U* = 35.50, *P* > 0.05) (Fig. 2D). This finding pointed to a symmetrical two-sided frequency range of the threat-selective generalization effect. We then proceeded to downshift the oscillation frequency of the NS+ by harmonically doubling the interval that separated it from the CS+. We repeated the fear conditioning and the recognition task (*n* = 12) with this new set of stimuli (CS−, 784 Hz; NS−, 1046 Hz; CS+, 370 Hz; NS+, 233 Hz) (Fig. 2E). In these conditions, we obtained a twofold discrimination (Fig. 2F,G). Participants succeeded in identifying both the nonreinforced (CS−, 87.5% versus NS−, 12.5%, *Z* = −2.99, *P* = 0.003) and the conditioned fear (CS+, 80% versus NS+, 20%, *Z* = −2.85, *P* = 0.004) stimuli, with no differences in the subjective confidence levels (CS− versus NS−, *U* = 28.50, *P* > 0.05; CS+ versus NS+, *U* = 37.50, *P* > 0.05) (Fig. 2H). These experiments showed a generalization of explicit recognition for stimuli perceptually close to but not perceptually distant from the CS+ and a lack of generalization to stimuli closely resembling the CS−. Therefore, the generalization of explicit responses was specifically connected with the aversive experience and occurred only with stimuli that may resemble the CS+.

### The generalization of explicit responses is not due to a degradation of memory processes

**Figure 3:**
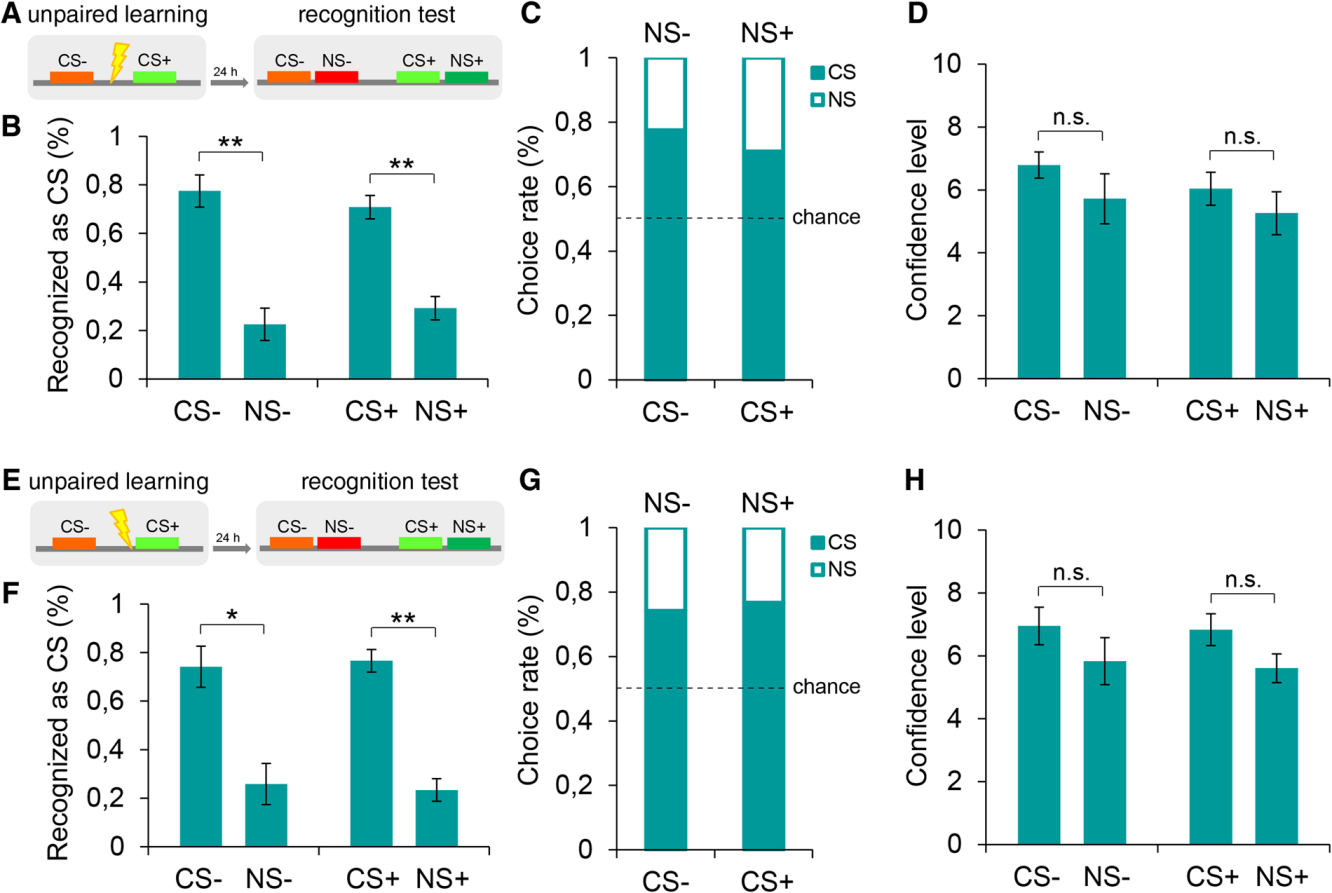
Recognition patterns after a stress-based unpaired learning. (**A**) Participants (*n* = 12) underwent an unpaired learning (CS+, 370 Hz; CS−, 784 Hz) in which painful stimuli pseudo-randomly occurred during the inter-trial-intervals, and not in association with a specific tone. Twenty-four hours later, subjects performed the 2AFC recognition task (NS+, 466 Hz; NS−, 1046 Hz). **(B,C)** Explicit detection levels were similarly high for both the CS− and the CS+, thus indicating that painful stimuli during learning trials did not interfere with encoding processes. **(D)** Subjects were similarly confident for both CSs recognition choices. **(E)** Participants (*n* = 12) encoded two tones (CS+, 370 Hz; CS−, 784 Hz) and painful stimuli were delivered 2 s prior to the CS+ onset and never before the CS−. This unpaired learning was aimed at reproducing the temporal proximity between painful stimuli and the CS+ without forming a CS-US predictive association. Twenty-four hours later subjects performed the explicit 2AFC test (NS+, 466 Hz; NS−, 1046 Hz). **(F,G)** Subjects highly recognized both the CS− and the CS+, thus indicating that the generalization of explicit responses is specifically related to the CS-US predictive association. **(H)** Confidence ratings did not differ between CS− and NS− or between CS+ and NS+ recognition choices. *P < 0.05, **P < 0.01, ***P < 0.001. All data are mean and SEM. Wilcoxon signed-rank tests [(**B,F**)]; Mann–Whitney U tests [(**D,H**)].

Our results led to the question of which factors contributed to the fear generalization we observed in the explicit recognition test. One possibility is that this form of generalization may be related to a stress-based interference on explicit associative processes that occur during learning. In fact, stressful stimuli, such as painful stimulations, might interfere with long-term memory^18–20^. Notably, the analysis of the SCRs revealed that, even if stress would have interfered with learning, it occurred only on the explicit processes, whereas it did not affect implicit memory. To better elucidate this point, we performed a control experiment during which painful stimuli were delivered at the same intensity as in previous experiments but not associated with a specific tone. Subjects were exposed to the identical sequence of two stimuli as in previous experiments, where one stimulus corresponded to the tone previously employed as CS+ (370 Hz) and the other stimulus corresponded to the tone previously adopted as CS− (784 Hz), and painful stimuli were delivered at pseudorandom intertrial intervals. On the test day, recognition patterns were collected by exposing subjects to the same sequence of tone-pairs composed of learned and novel stimuli similar to the CS+ (NS+, 466 Hz) or to the CS− (NS−, 1046 Hz) through the 2AFC task (Figs 3A and S1A). In the implicit test (*n* = 12), we found no significant differences in SCR levels among the four tones (χ^2^_(3)_ = 2.50, *P* > 0.05) (Fig. S1B). In the explicit test (*n* = 12), we found that the detection levels of the CS+ (70.8%) over the NS+ (29.2%) (*Z = *−2.77, *P* = 0.006) were high and comparable to that of the CS− (77.5%) over the NS− (22.5%) (*Z = *−2.58, *P* = 0.01) (Fig. 3B,C). The confidence ratings were similar for CS+ versus NS+ (*U* = 51.00, *P* > 0.05) and CS− versus NS− (*U* = 33.50, *P* > 0.05) (Fig. 3D). These data showed that a painful experience during stimuli encoding did not impair the discrimination of explicit responses. More importantly, these results suggested that the generalization of explicit responses, when present, may be related to the CS predictivity of the US. To test this possibility, we established an experimental paradigm in which painful stimuli were delivered concurrently with the CS+ but without allowing this tone to be predictive of the threat. We performed an experiment (*n* = 12) in which the delivery of the shock was set 2 s prior to the CS+ onset while it never preceded the CS− (Fig. 3E). In the 2AFC recognition test, subjects mostly identified the CS+ (76.7%) over the NS+ (23.3%) stimulus (*Z = *−2.95, *P* = 0.003) as well as the CS− (74.2%) over NS− (25.8%) (*Z = *−2.16, *P* = 0.031) (Fig. 3F,G). Additionally, in this case, there were no significant differences in confidence levels (CS− versus NS−, *U* = 30.00, *P* > 0.05; CS+ versus NS+, *U = *36.50, *P* > 0.05) (Fig. 3H). Taken together, these findings suggested that any stress-related effect due to the administration of the painful stimuli did not interfere with the ability to encode the CS+. More importantly, these data revealed that the generalization of explicit fear responses, when present, was specifically related to associative processes connecting sensory stimuli to threatening events.

### The generalization of explicit fear responses is driven by the valence of the stimulus and the learning context

**Figure 4:**
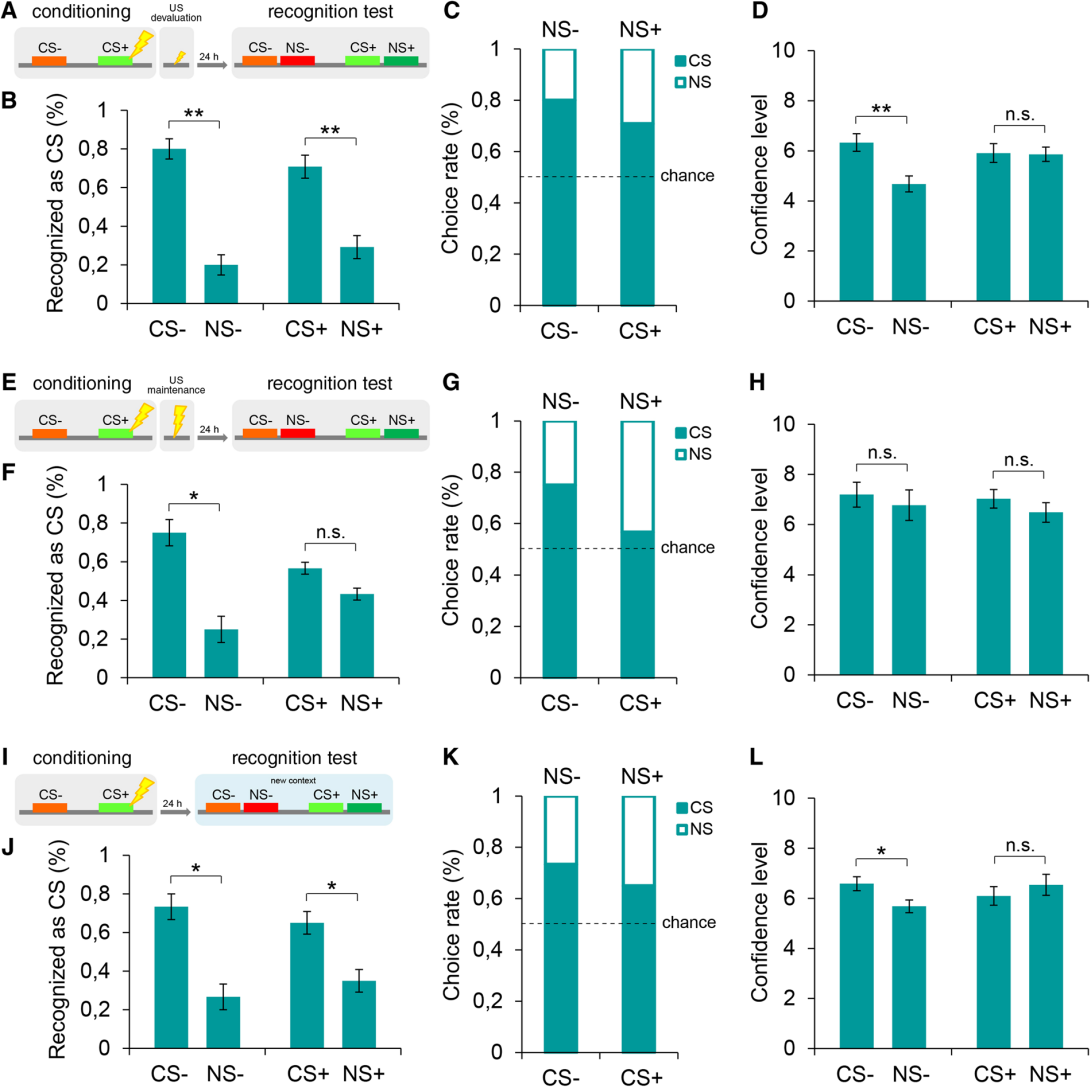
The role of stimulus-embedded valence and learning context in driving the generalization of explicit threat responses. (**A**) Shortly after the threat conditioning (CS+, 370 Hz; CS−, 784 Hz), participants (*n* = 12) were repeatedly administered with solely USs whose intensity was lower than in the conditioning phase. This procedure was aimed at devaluating the threatening value embedded into the CS+. Twenty-four hours later, they performed the 2AFC recognition task (NS+, 466 Hz; NS−, 1046 Hz). **(B,C)** In the explicit recognition task subjects successfully identified both the CS− and the CS+, thus indicating that devaluating the fearful outcome predicted by the CS+ limits the generalization of explicit responses. **(D)** Confidence judgments were higher for correct than incorrect responses for the CS− but not for the CS+. **(E)** In the control condition, participants (*n* = 12) were repeatedly exposed to USs whose intensity was kept constant from the conditioning phase. **(F,G)** Controls explicitly recognized the CS− but generalized the identification of the CS+ to the NS+, thus showing the threat-specific generalization pattern. **(H)** Confidence levels did not differ for both CSs recognition choices. **(I)** Twenty-four hours after the threat conditioning (CS+, 370 Hz; CS−, 784 Hz), subjects (*n* = 12) performed the explicit 2AFC test (NS+, 466 Hz; NS−, 1046 Hz) within a new physical context which was located inside of another building. **(J,K)** Participants exhibited a successful recognition of both the CS− and the CS+, thus indicating that encountering the CS+ in a different environment from the conditioning phase shifted the explicit threat-recognition pattern from generalization (old context) to discrimination (new context). **(L)** Participants were more confident when identifying than when misidentifying the CS− but not the CS+. *P < 0.05, **P < 0.01, ***P < 0.001. All data are mean and SEM. Wilcoxon signed-rank tests [(**B,F,J**)]; Mann–Whitney U tests [(**D,H,L**)].

The data collected thus far suggested that the generalization of explicit responses may have been specifically connected to a function dedicated to the prediction of danger. To clearly assess this possibility, we performed a series of experiments. First, we tested whether the generalization depended on the threatening value of the CS+. To this end, we probed whether a postlearning manipulation of the CS-paired emotional content interfered with the observed generalization of the explicit responses. We adopted the postconditioning US devaluation technique^21^, which allows modulation of the valence of the US representation without affecting the learned CS-US association^22^. This procedure consisted of attenuating the aversiveness of the US, after the CS-US pairings, through the delivery of shocks alone at a lower intensity than that employed during the conditioning. Shortly after the threat learning (~1–2 min), one group of participants (devaluation group, *n* = 12) was repeatedly administered the US at a lower intensity (Fig. 4A), while another group of participants (control group, *n* = 12) received the US alone at the same intensity as in the conditioning phase (Fig. 4E). To validate the effectiveness of this procedure in devaluating the US representation, subjects were required to evaluate the aversiveness of the US on the same analog scale used in the pre- and post-conditioning phases. We obtained significantly lower ratings in the devaluation group (*Z* = −3.08, *P* > 0.002) but not in the control group (*Z* = −1.81, *P* > 0.05) than those provided after the conditioning phase (see Table S1). The day after this procedure, both groups were tested in the 2AFC recognition task. Critically, the devaluation group successfully detected the CS+ (70.8%) over the NS+ (29.2%) (*Z = *−2.57, *P* = 0.01), as well as the CS− (80%) over the NS− (20%) (*Z = *−2.95, *P* = 0.003) (Fig. 4B,C). In contrast, the control group displayed the threat-specific generalization pattern that we observed earlier by similarly recognizing the CS+ (56.7%) and the NS+ (43.3%) as the CS (*Z = *−1.93, *P* > 0.05) and correctly identifying the CS− (75%) and not the NS− (25%) as the CS (*Z = *−2.55, *P* = 0.011) (Fig. 4F,G). In the devaluation group, confidence levels were higher for correct than incorrect responses for the nonreinforced (*U* = 17.00, *P* = 0.007) but not for the threatening (*U* = 58.00, *P* > 0.05) stimulus (Fig. 4D). In the controls, there were no differences in confidence levels (CS− versus NS−, *U* = 39.00, *P* > 0.05; CS+ versus NS+, *U = *49.00, *P* > 0.05) (Fig. 4H). These data demonstrated that decreasing the threatening value of the US after the conditioning prevented the explicit generalization that we had otherwise observed. Thus, the generalization of explicit responses may have stemmed from an adaptive and flexible function of the explicit system that might be able to actively generalize an explicit stimulus representation based on its learned threatening valence. If a subsequent experience reshapes this meaning, this mechanism updates its predictions and may actively limit the generalization process.

To examine the flexible nature of this predictive mechanism in depth, we sought to probe whether this mechanism was also influenced by the environmental context where the threatening experience has occurred. In fact, the surrounding context may shape and define the perception of sensory traces and memories of past episodes^23^. In particular, we were interested in whether a shift in context may change the prediction of the danger-related mechanism and modulate the level of generalization. To address this idea, one group of participants (*n* = 12) underwent the same conditioning procedure as in the previous experiments, but on the following day, the 2AFC explicit task was performed in a completely different environment (Fig. 4I). In this new situation, subjects detected the CS+ (65%) over the NS+ (35%) (*Z* = −2.07, *P* = 0.039) and the CS− (73.3%) over the NS− (26.7%) (*Z* = −2.50, *P* = 0.013), thus indicating a successful recognition of the nonreinforced as well as the threatening stimuli (Fig. 4J,K). Participants were more confident with correct than incorrect responses for the nonreinforced stimulus but not for the threatening one (CS− versus NS−, *U* = 33.00, *P* = 0.044; CS+ versus NS+, *U* = 57.50, *P* > 0.05) (Fig. 4L). This result indicated that when the individuals were placed in a context that was different from that where the aversive experience had occurred, they did not show generalization of the explicit responses. This evidence is consistent with the idea that the explicit identification of a learned threat also depends on the cognitive evaluation of contextual sensory cues. Overall, these data supported the idea that the explicit generalization was related to a cognitive mechanism that was able to predict the occurrence of danger in a flexible manner by using information about the embedded emotional content and the surrounding context where the threatening experience occurred.

## Discussion

In this study, we investigated whether the implicit and explicit response patterns to novel stimuli overlapped or diverged after learning about a threat. By using discriminative auditory fear conditioning, we found a striking dissociation between implicit and explicit threat recognition patterns. The evoked autonomic responses, analyzed by SCRs, were strong and selective in response to the CS+ and weak in response to the NS+, the CS− and the NS−. Conversely, the explicit recognition profile revealed that subjects were highly prone to misidentify the NS+ as the encoded CS+, thus showing a generalization of responses that approximated the chance level. Critically, the CS− was explicitly recognized and correctly discriminated from the NS−. Moreover, the generalization phenomenon did not occur when the NS+ was very different from the CS+.

These findings suggest that implicit reactions are highly sensitive to detecting a cue learned as dangerous, whereas explicit responses are more inclined to express a generalization pattern that is selective for the threat stimuli and does not extend to safe stimuli. Hence, implicit and explicit profiles are dissociable and seem to not exert any reciprocal influence when encountering a new stimulus that reminds an individual of a learned threat.

A possible alternative interpretation of our data is that the generalization of explicit responses may be due to a degradation in memory processing induced by stress-related interference during the learning trial. Previous studies have found that exposure to a stressor affects long-term memory^18–20^. To disentangle between these possibilities, we performed two experiments in which the painful stimuli were delivered independent of a specific stimulus, and we found that participants explicitly discriminated between the learned threatening stimuli and the novel stimuli. Thus, the generalization of explicit recognitions, when present, was related to the learned CS-US association and not due to a degradation of memory processes. These data led us to test whether the generalization of explicit reactions may have represented a predictive mechanism dedicated to inferring the potential danger of incoming stimuli. Through a US devaluation procedure^21^ and a context shift from the learning to the testing phase, we showed that this form of generalization is driven by the valence of the threatening stimulus and by contextual sensory cues.

Previous studies^22,24^ have found an attenuating effect of US devaluation on conditioned implicit responses but a lack of effect on explicit expectancy ratings. Here, we did not adopt an expectancy-based task, but we adopted a recognition-based task to test the explicit response pattern. In other words, we did not investigate how much subjects expected the US when exposed to the CSs and the NSs because this index would have not allowed a proper differentiation of the CS− from the NS−. Rather, we tested the ability to explicitly recognize both CSs and distinguish them from the NSs. To the best of our knowledge, whether and how a modification in the US representation affects the explicit recognition of the CS+ had never been investigated before.

We also found that testing subjects in a context different from that of the conditioning phase enabled the discrimination between the CS+ and the NS+. In line with our results, previous studies showed that conditioned responses can be triggered by sensory stimuli and the surrounding context paired to aversive events^25^. Indeed, contexts may be useful to disambiguate uncertain situations to guide adaptive behavior^23^. A context-dependent specificity effect in fear conditioning was found in an earlier human study where implicit cued fear to the CS+ compared to the CS− was evident when subjects were tested in the same conditioning context and not when tested in a novel context^26^.

Concerning the specificity of the explicit generalization, previous studies^27–30^ have indicated that sensory acuity is shaped following aversive learning. This effect is thought to be due to the plastic modification of sensory discrimination thresholds (^28,30–32^; but see^33,34^). This effect is unlikely to explain our data since we adopted stimuli that were highly perceptually discriminable at both the explicit and implicit levels. In our experiments, therefore, participants seemed to explicitly respond on the basis of a generalized representation of the threatening stimulus.

It has been previously reported that fear reactions at explicit (US-expectancy) and implicit (SCR) levels were a reflection of a perceptual continuum, even if the reactions are predicted by distinct patterns of neuronal tuning in the anterior insula (aIC) and the inferotemporal cortex (ITC)^5^. Our results provide new evidence on how fearful recognition at the behavioral level may not be mirrored in a defensive response at the autonomic level, thus adding further complexity to the circuits involved in threat detection processes. Hence, these data are inconsistent with the idea that fear generalization arises from a unitary implicit-explicit process. Rather, the data support the idea that the source of fear generalization is related to multiple systems, where the explicit responses to stimuli resembling a learned threat do not influence nor are influenced by the implicit processes. These differential patterns indicate that these systems may activate dissociable reactions towards the same stimulus. These observations seem to be in line with a recently proposed two-system framework^35^ that includes a defensive survival circuit, supporting behaviors and physiological reactions to threats, and a cognitive circuit, supporting subjective experiences of fear, and may be dissociable.

Several lines of evidence have shown that dissociable neural systems encode the implicit and explicit memories of a threatening experience^11,12^. Dissociations between implicit and explicit learning in threat conditioning have been found in brain-damaged patients^36^, in studies reporting implicit fear learning in the absence of explicit contingency awareness^37–40^, during implicit fear discrimination for consciously indiscriminable stimuli^41,42^ and in fear reduction through a procedure that eludes conscious exposure to the learned threat^43,44^. In humans, nonhuman primates and rats, many brain regions have been identified as involved in implicit fear generalization and discrimination processes^1,45^, such as the prefrontal and auditory cortices^46–50^, the amygdala^51,52^, and the insular cortex^5,53^. However, explicit associative recognition relies on the hippocampal and medial temporal lobe (MTL) networks^54^. An earlier study found that rats with a lesion to the hippocampus showed better fear discrimination^55^. The authors hypothesized that the generalization within different memory systems may exhibit distinct gradients, where the hippocampus may be less finely tuned than other systems (such as thalamo-amygdala and thalamo-cortico-amygdala memory systems). This divergence may be due to the hippocampus’s role in extracting generalities from associative threatening experiences^56–60^. Thus, hippocampal processing may result in a generalized response^55^. Our findings in humans seem to support this hypothesis by providing evidence that individuals may tend to generalize the explicit identification of a learned threat even when they are capable of implicitly detecting differences.

The relevance of these results also applies to the potential dysregulation of these flexible mechanisms in PTSD and fear-related disorders. Traumatic events may affect the cognitive and highly adaptive explicit evaluation of incoming stimuli, such as the context-dependent shift or the ability to actively limit the generalization process that we have described here, thereby leading to dysfunctional cognitive predictions. Previous studies have highlighted the relevance of context information processing for fear-related disorders, such as PTSD or panic disorder^61–63^. Indeed, a dysregulation in the ability to contextualize information may lead to inaccurate percepts, improper attributions of meaning to stimuli and rigid behaviors^23^. As such, PTSD patients show a markedly greater contextual fear than non-PTSD individuals^64,65^ and exhibit a compromised ability to use contextual information to limit defensive responses to stimuli that are no longer predictive of aversive consequences^66^.

In conclusion, our findings reveal a divergence between the implicit and the explicit tunings in threat identification processes. Moreover, they highlight the operations of a cognitive mechanism that is able to flexibly evaluate incoming stimuli to develop adaptive predictions of potential dangers.

## Methods

### Participants

All participants (*n* = 176) were healthy university students (mean age: 21.61 ± 2.49 S.D., 50 males and 126 females) with no history of psychiatric disorders, neurological illnesses, cardiovascular diseases and illegal drug use. During the pre-experimental screening phase, each participant was also administered with the *State-Trait Anxiety Inventory Form Y*^67,68^. Participants who showed a score >80 in the sum of the two subscales (State + Trait anxiety) were not included in the sample. Musicians and individuals who reported a past or current musical training were not included in the sample (see Table S1 for all groups’ mean age and State-Trait Anxiety Inventory scores). After this preliminary phase, participants were randomly assigned to each experimental condition. We discarded five participants because of excessively low SCRs, and three participants because they did not understand the task, leaving a total of 168 participants. Each participant provided written informed consent after receiving a complete description of the experimental procedures. All experimental procedures were performed in accordance with the ethical standards of the Declaration of Helsinki and were approved by the Bioethics Committee of the University of Turin.

### Auditory stimuli

Auditory stimuli were pure sine wave tones with oscillation frequencies of 233 Hz, 294 Hz, 370 Hz, 466 Hz, 784 Hz and 1046 Hz, lasting 6 s with onset/offset ramps of 5 ms. Tones were digitally generated using Audacity 2.1.2 software (Audacity® freeware), and binaurally delivered through headphone speakers (Beyerdynamic DT770 Pro) at ~50 dB intensity. Experiments were conducted in a dimly lit room, and all experimental scenarios were controlled by Presentation® 17.2 software (NeuroBehavioral Systems, Berkeley, CA).

### Two-alternative forced-choice (2AFC) perceptual discrimination test

The task consisted in comparing 20 pairs of auditory stimuli (370 Hz, 466 Hz, 784 Hz and 1046 Hz) which were presented with a 1000-ms intra-pair-interval in a pseudorandom sequence (inter-pair-interval of 24 s). For each pair, subjects were asked to refer whether the two tones were “the same tone or different tones”, and to provide a confidence rating on an analog scale from 0 (completely unsure) to 10 (completely sure). No feedback was supplied.

### Unconditioned stimulus calibration procedure

Before starting with the calibration procedure, systolic and diastolic blood pressure was measured in order to prevent possible hypoarousal reactions caused by a basal hypotension. The unconditioned stimulus (US) consisted of a mild electrical shock (train pulse at 50 Hz lasting 200 ms, with a single pulse duration of 1000 µs) generated with a direct current stimulator (DS7A Constant Current Stimulator, Digitimer). Impulses were delivered through a bar stimulating electrode connected by a Velcro strap on the upper surface of the dominant hand’s index finger. The electrical stimulation intensity was individually calibrated through a staircase procedure^69^, starting with a low current near the perceptible tactile threshold (~0.5 mA). Participants were asked to rate the aversiveness of each train-pulse on a scale ranging from 0 (not painful at all), 1 (pain threshold) to 10 (highly painful if protracted in time). At the end of the procedure, the US amplitude was set at the current level (mA) corresponding to the mean rating of ‘7’ on the subjective analog scale.

### Pre-conditioning habituation

This phase followed the US calibration and consisted in the presentation of 4 stimuli: 2 CS− (784 Hz) and 2 CS+ (370 Hz) tones with an inter-trial-interval (ITI) of 24 s, in absence of any US. In the experiment showed in Fig. 1F–J, the frequencies of tones were inverted (i.e. 370 Hz served as CS− and 784 Hz served as CS+). At the end of this phase, participants were asked to confirm whether the tones were easily audible but not too loud or annoying.

### Conditioning and unpaired learning

#### Conditioning

After a 5-min resting period, participants underwent a discriminative fear conditioning, which consisted in the presentation of 30 stimuli: 15 CS+ (370 Hz) and 15 CS− (784 Hz) in a pseudorandom sequence, with an inter-trial-interval (ITI) of 24 s. The CS+ co-terminated with the US 12 times (80% reinforcement rate), while the CS− was never paired with the US. In the experiment showed in Fig. 1F–J, the frequencies of tones were inverted (i.e. 370 Hz served as CS− and 784 Hz served as CS+).

#### Unpaired learning

After 5 min of resting, participants underwent the identical pseudorandom sequence of tones (CS+: 370 Hz, CS−: 784 Hz) as in the paired conditioning protocol, but the US was not associated to an auditory stimulus. In the unpaired learning (Fig. 3A), 12 USs pseudo-randomly occurred during the 24-s ITI at 9 s, 12 s or 15 s from the tone offset. In the concurrent unpaired learning (Fig. 3E), the US was delivered 2 s before the CS+ onset with a 80% scheduling (i.e., for 12 times the CS+ started 1.8 s after the 200-ms shock offset) and never preceded the CS−.

In all acquisition procedures, subjects were not informed about any possible CS-US contingency. To validate the emotional learning, immediately following this phase subjects rated the aversiveness of the US using the same analog scale as in the pre-conditioning calibration procedure (see Table S1 for all groups’ US current intensity and US analog ratings).

### Unconditioned stimulus devaluation

Immediately after the conditioning session (~1–2 min), subjects were administered with 6 US trials in absence of tones (1-ms pulses, 50 Hz, train duration 200 ms) with a 29.8 s inter-trial-interval (reproducing the exact time-pattern of shock delivery as in the conditioning phase). Participants were not informed about any possible modification in the electrical stimulation intensity. In the devaluation group, the US amplitude (mA) was decreased to the mean rating of ‘2’ on each participant’s subjective scale. In the control condition, the US amplitude was maintained constant as in the conditioning phase. At the end of the procedure, each participant was asked to give a post-revaluation rating of the US aversiveness on the subjective scale (See Table S1).

### Two-alternative forced-choice (2AFC) recognition test

This procedure involves the presentation of two stimuli on each trial and was preferred over a yes-no paradigm (which involves one stimulus on each trial and a new/old judgment task) since it improves performance and discourages response bias in a recognition memory task, such as a familiarity-based decision bias^17^.

Throughout all the testing phases, the stimulating electrode was kept attached as in the conditioning phase in order to create the expectation to receive the US. Differently from other generalization paradigms which involve the delivering of the US in order to prevent extinction^3,5,13,42^, here no shocks were delivered, i.e. the CS+ was never paired with the US, in order to avoid any reacquisition effect.

After a 5-min resting period, participants underwent a two-alternative forced-choice (2AFC) task, which consisted in the presentation of 20 pairs of auditory stimuli, each composed by a conditioned stimulus (CS− or CS+) and a new stimulus similar to the CS− (NS−, 1046 Hz in Figs 1A–E, 2, 3 and 4; 466 Hz in Fig. 1F–J) or to the CS+ (NS+, 466 Hz in Figs 1A–E, 3 and 4; 1046 Hz in Fig. 1F–J; 294 Hz in Fig. 2A–D; 233 Hz in Fig. 2E–H) in a pseudorandom sequence: 5 × CS− vs NS−, 5 × NS− vs CS−, 5 × CS+ vs NS+, 5 × NS+ vs CS+. On each trial, the two stimuli were presented with an intra-trial-interval of 1000 ms. After 5 s from the pair offset, a 60-s auditory interference (see next section) and a 24-s silent ITI occurred. In the implicit test, SCRs were recorded throughout this phase. In the explicit test, participants were explained that in each couple of sounds there was a tone that they had heard on the day before, and a new tone. Participants were then instructed to recognize and verbally refer which one (the first or the second) was the tone heard on the day before, paired (CS+) or not paired (CS−) with the US-shock. Participants were further asked to verbally provide a confidence rating about each response, on a scale from 0 (completely unsure) to 10 (completely sure). No feedback was supplied. In the experiment showed in Fig. 4I–L, this procedure was performed in a context different from that of the conditioning phase. This new setting consisted in room whose sensory features were highly dissimilar from those of the conditioning room, and which was located inside of another building. Notably, in the new environment subjects still wore the electrode, an important predictor signaling the possible occurrence of a shock^25^.

### Auditory working memory interference

When hearing a serial sequence of tones subjects can actively take advantage of a pitch comparison mechanism due to the auditory working memory (AWM) rehearsal process^70,71^. In our testing protocol, if participants were not prevented from rehearsing during the inter-trial-interval, each response (except for the first one) might be affected by the sensory comparison of each pair of tones with the previous one in the sequence, thus introducing cognitive biases in the recognition process. Given that a method to interfere with the rehearsal process is filling the inter-trial-interval with a series of additional tones^72,73^, we created an auditory retroactive interference in order to prevent possible cognitive biases during the recognition test. The interference consisted in 60 s of 10-s mixed samples of pop music.

### Psychophysiological recording and analysis

Event-related skin conductance responses (SCRs) were used as an implicit index of fear responses. In order to record the autonomic signal, two Ag-AgCl non-polarizable electrodes filled with isotonic paste were attached to the index and middle fingers of the non-dominant hand by Velcro straps. The transducers were connected to the GSR100C module of the BIOPAC MP-150 system (BIOPAC systems, Goleta, CA) and signals were recorded at a channel sampling rate of 1000 Hz. SCR waveforms were analyzed offline using AcqKnowledge 4.1 software (BIOPAC systems, Goleta, CA). Each SCR was evaluated as event-related if the trough-to-peak deflection occurred 1–6 s after the stimulus onset, the duration was comprised between 0.5 and 5.0 s, and the amplitude was greater than 0.02 microsiemens (μS). Responses that did not fit these criteria were scored as zero. Because the implicit test was configured as 2AFC with a 1-s ITI, the range of the analysis was restricted to 1–7 s following the onset of the first stimulus of the pair. That is, given the sequence of 6 s (1^st^ tone), 1 s (intra-trial-interval) and 6 s (2^nd^ tone) which defined the structure of each pair, a temporal cut-off was established upon the onset of the second stimulus of the pair, in order to avoid summing effects in the event-related responses. Raw SCR data of each subject were standardized through a Z-score transformation^74,75^ and averaged by stimulus type (CS−, NS−, CS+, NS+).

### Statistical analyses

Since several variables did not pass the Shapiro-Wilk test for the normality of the distribution, non-parametric statistics were adopted in each experiment. Friedman test followed by Dunn’s *post-hoc* multiple comparison tests were used for autonomic data analyses. Explicit recognition choice rates, perceptual judgments and US ratings were analyzed by performing Wilcoxon signed-rank tests. In the analysis of confidence ratings, participants who exhibited a perfect (100%) recognition of one or both CSs in the 2AFC task generated missing cells for the respective NS or NSs tones. Because a paired comparison would have excluded all the confidence judgments of these participants, we performed unpaired Mann-Whitney U tests in order to include all the available data in the analysis. In order to compute the correlation between STAI-Y scores and implicit/explicit recognition levels, the Spearman’s rank correlation coefficient was adopted. The null hypothesis was rejected at *P* < 0.05 significance level. All statistical analyses were performed using SPSS Statistics 22 (IBM) and Prism 6.05 (GraphPad).

## Supplementary information

Supplementary Information
