# Supplementary material for: Implicit and explicit systems differently predict possible dangers
Source: Sci Rep. 2019 Sep 16;9:13367. doi: 10.1038/s41598-019-49751-4 (PMC6746769; doi:10.1038/s41598-019-49751-4)
Supplement: Supplementary file 1 — Supplementary Information [file 41598_2019_49751_MOESM1_ESM.pdf]

Implicit and explicit systems differently predict possible dangers

Eugenio Manassero, Ludovica Mana, Giulia Concina, Annamaria Renna and Benedetto Sacchetti

Supplementary Information

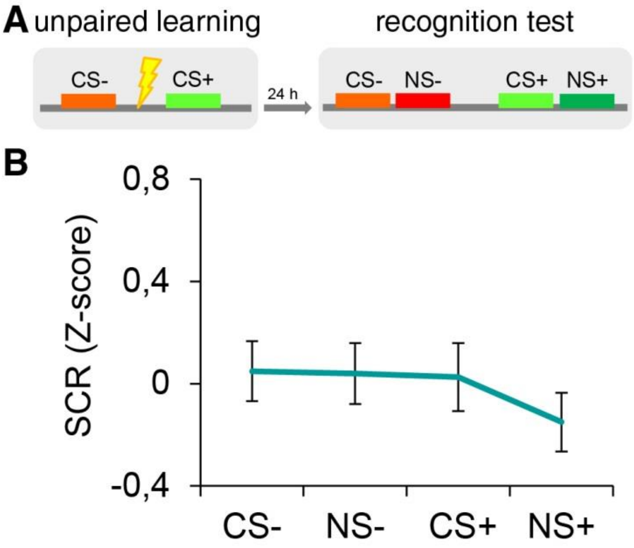

Figure S1. Implicit response pattern after a stress-based unpaired learning (A) Schematic diagram depicting the experimental procedures. Participants (*n* = 12) underwent an unpaired learning (CS+, 370 Hz; CS-, 784 Hz) in which painful stimuli were delivered during the inter-trial intervals, not in association with a specific tone. Twenty-four hours later, subjects performed the implicit 2AFC task (NS+, 466 Hz; NS-, 1046 Hz). **(B)** Implicit recognition profiles demonstrated a lack of discrimination, as subjects similarly responded to all tones. * $P < 0.05$, ** $P < 0.01$, *** $P < 0.001$. All data are mean and SEM. Friedman test, non-significant [(B)].

Table S1. Experimental groups' anagraphical and procedural details

Experimental group	Sample width	Age	STAI-Y State	STAI-Y Trait	STAI-Y Total	Conditioning US current intensity (mA)	Post-conditioning US analog rating	Revaluation US current intensity (mA)	Post-revaluation US analog rating
Implicit task	*n* = 18	20.75 ± 2.47	33.83 ± 2.81	39.00 ± 5.56	72.83 ± 6.49	4.15 ± 2.33	5.69 ± 1.21	-	-
Explicit task	*n* = 18	25.52 ± 3.32	30.89 ± 6.61	33.22 ± 6.08	64.11 ± 10.96	4.40 ± 1.79	5.67 ± 1.18	-	-
Implicit task inverted tone frequencies	*n* = 18	21.60 ± 1.84	31.17 ± 6.04	37.89 ± 6.34	69.06 ± 10.16	4.67 ± 2.02	5.86 ± 1.16	-	-
Explicit task inverted tone frequencies	*n* = 18	20.57 ± 1.77	32.33 ± 4.04	36.83 ± 4.05	69.17 ± 5.61	5.24 ± 3.96	5.25 ± 1.22	-	-
Explicit task symmetrically lower-pitched NS+	*n* = 12	21.41 ± 1.90	29.50 ± 3.55	35.25 ± 5.48	64.75 ± 8.36	5.01 ± 1.90	5.63 ± 1.03	-	-
Explicit task distantly lower-pitched NS+	*n* = 12	20.57 ± 1.60	34.08 ± 5.92	36.25 ± 5.22	70.33 ± 8.50	5.67 ± 2.89	6.46 ± 1.34	-	-
Implicit task unpaired learning	*n* = 12	21.64 ± 2.23	30.08 ± 3.73	35.92 ± 6.44	66.00 ± 7.95	4.25 ± 2.63	5.96 ± 0.75	-	-
Explicit task unpaired learning	*n* = 12	20.43 ± 1.15	30.67 ± 5.43	37.50 ± 5.87	68.17 ± 7.88	4.28 ± 2.01	6.00 ± 1.35	-	-
Explicit task concurrent unpaired learning	*n* = 12	20.80 ± 1.16	33.58 ± 2.57	36.58 ± 4.96	70.17 ± 6.52	3.35 ± 1.25	5.42 ± 1.28	-	-
Explicit task US devaluation	*n* = 12	21.68 ± 1.44	32.75 ± 3.55	39.33 ± 5.48	72.08 ± 8.08	4.97 ± 2.27	5.08 ± 1.16	1.81 ± 0.60	2.08 ± 0.97
Explicit task US maintenance (controls)	*n* = 12	21.70 ± 2.31	31.00 ± 5.92	36.83 ± 6.24	67.83 ± 11.72	3.91 ± 1.63	5.13 ± 1.17	3.91 ± 1.63	5.71 ± 1.57
Explicit task context-switch	*n* = 12	22.55 ± 2.43	31.58 ± 5.16	34.92 ± 6.42	66.50 ± 9.37	4.15 ± 1.37	5.42 ± 0.93	-	-

Notes: All data are mean ± SD

Table S2. Correlation matrices of threat recognitions and STAI-Y scores

	Implicit task (*n* = 18)			Implicit task inverted tone frequencies (*n* = 18)	
	**STAI-Y state**	**STAI-Y trait**		**STAI-Y state**	**STAI-Y trait**
**CS+**	0.351	-0.178	**CS+**	-0.249	-0.402
**NS+**	-0.194	0.215	**NS+**	-0.100	-0.055
	**Explicit task (*n* = 18)**			**Explicit task inverted tone frequencies (*n* = 18)**	
	**STAI-Y state**	**STAI-Y trait**		**STAI-Y state**	**STAI-Y trait**
**CS+**	0.017	0.047	**CS+**	-0.105	-0.166
**NS+**	-0.017	-0.047	**NS+**	0.105	0.166

Notes: all Spearman's rho correlations are non-significant
